# Impact of g force and timing on the characteristics of platelet-rich fibrin matrices

**DOI:** 10.1038/s41598-021-85736-y

**Published:** 2021-03-16

**Authors:** Ana B. Castro, C. Andrade, X. Li, N. Pinto, W. Teughels, M. Quirynen

**Affiliations:** 1 Department of Oral Health Sciences, Periodontology, KU Leuven and Dentistry, University Hospitals Leuven, Kapucijnenvoer 7, blok a - bus 07001, 3000 Leuven, Belgium; 2 Department of Periodontology and Oral Implantology, Faculty of Dentistry, School of Dentistry, University of Los Andes, Santiago, Chile; 3 Department of Oral Health Sciences, KU Leuven, BIOMAT and University Hospitals Leuven Dentistry, Leuven, Belgium

**Keywords:** Biomedical materials, Tissue engineering

## Abstract

Recently, new centrifugation protocols for the preparation of platelet-rich fibrin (PRF) have been introduced in an attempt to further improve the beneficial impact of these 2nd generation platelet concentrate membranes. This in-vitro study aimed to compare the biological and physical characteristics of three types of PRF membranes using two different centrifuges with adapted relative centrifugal forces (RCF): leucocyte- and platelet-rich fibrin, advanced platelet-rich fibrin, and advanced platelet-rich fibrin^+^. Release of growth factors, macroscopic dimensions, cellular content and mechanical properties of the respective membranes, prepared from blood of the same individual were explored. Furthermore, the impact of timing (blood draw-centrifugation and centrifugation-membrane preparation) was assessed morphologically as well as by electron microscopy scanning. No statistically significant differences amongst the three PRF modifications could be observed, neither in their release of growth factors or the cellular content, nor in clot/membrane dimensions. The difference between both centrifuges were negligible when the same *g*-force was used. A lower *g*-force, however, reduced membrane tensile strength. Timing in the preparation process had a significant impact. Adaptation of RCF only had a minimal impact on the final characteristics of PRF membranes.

## Introduction

A centrifuge creates a centrifugal force for separating substances of different densities in a liquid by rotating at a certain speed measured as revolutions per minute, RPM. The force applied during centrifugation is called relative centrifugal force (RCF)^1^. It causes denser substances and particles to move outward in the radial direction. Denser particles thus settle at the bottom of the tube, while low-density substances move to the top^2^. This technique is often used to separate red blood cells from serum or plasma. Based on this procedure, the concept of platelet concentrates (PCs) arose in the ’70s^3^, and in the late ‘90 s and beginning of the 00`s their use gained more interest in the oral and maxillofacial field^4,5^. In 2009, PCs were newly classified into four categories depending on leucocyte inclusion and architecture^6^: pure platelet-rich plasma (P-PRP), leucocyte and platelet-rich plasma (L-PRP), pure platelet-rich fibrin (P-PRF), and leucocyte- and platelet-rich fibrin (L-PRF). L-PRF is obtained after centrifugation of blood in glass or silica-coated plastic tubes without the use of anticoagulants, such as EDTA. Three layers are obtained: red blood cells at the bottom, a buffy coat (clot) consisting of leucocytes and platelets in the middle, and a-cellular plasma at the top. The original protocol for L-PRF provided centrifugation at RCF_clot_: 408 g (RCF_max_: 653 g, RCF_min_: 326 g, RCF_average_: 489 g, distance to rotor for RCF_clot_: 50 mm) for 12 min in order to reach high concentrations of platelets and leucocytes in the buffy coat^7^. In the past decade, the use of L-PRF has increased exponentially^8,9^.

Separating substances of different densities by centrifugation depends on several aspects, including speed (rotation/revolutions per minute), and duration of spinning. The *g-* force is influenced by the angulation and radius of the rotor in the centrifuge and these differ widely depending on the type of centrifuge^10^. Rotor stability also has a significant impact, with reduced separation in case of radial vibration. In any evaluation or comparison of medical devices and protocols in this area, factual accuracy is of the utmost importance. Even centrifugation at identical RPM will exert different centrifugal forces if centrifuge rotors have different radius sizes, bucket types or bucket sizes. In 2014, Ghanaati and co-workers^11^ proposed a new protocol increasing the time of centrifugation and decreasing speed (A-PRF, RCF_clot_ 193 g, RCF_max_: 276 g for 14 min), using glass tubes for blood collection. Recently, the same group introduced another modification^12^ by reducing centrifugation speed and duration even further (A-PRF + , RCF_clot_ 145 g, RCF_max_: 208 g for 8 min). Reducing RCF resulted in an increase in the release of growth factors and in the concentration of leucocytes and platelets.

Studies comparing A-PRF or A-PRF + with L-PRF have led to controversial data^12–14^. For instance, Ehrenfest and co-workers^15^ compared L-PRF versus A-PRF prepared with various centrifugation devices and concluded that the L-PRF protocol allowed producing larger clots/membranes and a more intense release of growth factors. In contrast, in a similar study El Bagdadi and co-workers^13^ compared L-PRF versus A-PRF versus A-PRF + and observed an increased in growth factors release when RCF was reduced. Comparing findings is complicated by the heterogeneity in methods used, such as type of tube (plastic or glass) and adaptation of RCF to the rcf-max or rcf-clot. Moreover, neither of these studies evaluated the real effect of the centrifuge when the same PRF matrices were prepared with the *g* force adapted for each device nor the impact of using a glass or plastic tube.

Therefore, the primary aim of this study was to investigate whether the adaptation of the *g* force for the above-mentioned PRF modifications (L-PRF, A-PRF, and A-PRF +) in 2 centrifuges have any influence on their characteristics in terms of release of growth factors, morphology, cellular content, and mechanical properties. Although speed and duration of centrifugation are crucial, timing of the entire process also appears to be an important factor. Therefore, the secondary aim was to assess the influence of time before and after centrifugation on L-PRF membrane morphology.

## Results

Eight healthy subjects (4 women, 4 men) participated in this study. The mean age was 42.8 ± 14.2 years (range 29–60 years). No complications during blood collection were reported. In one subject, the L-PRF-DUO membrane was completely dissolved at the 7–14 day time interval. The same occurred with the A-PRF + -IL of another subject at the same time interval.

### Comparison of PRF modifications

#### Release of growth factors per time interval (Fig. 1*)*

**Figure 1:**
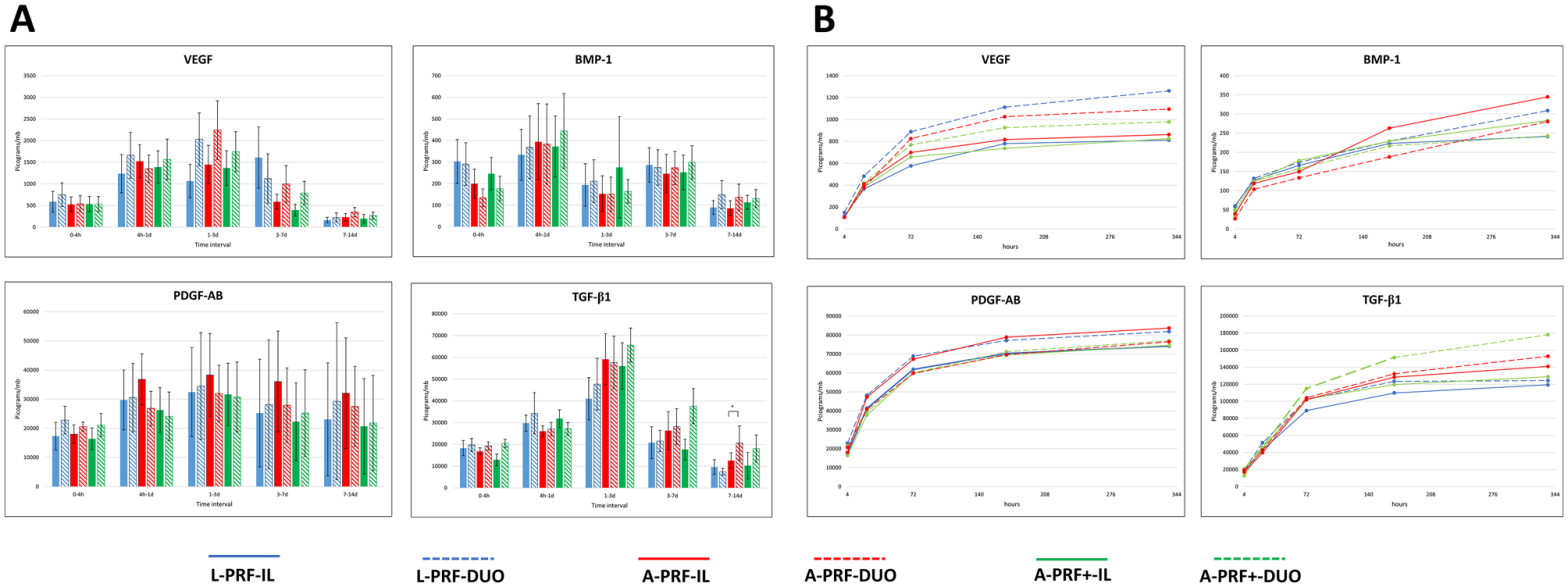
Release of growth factors (VEGF, BMP-1, PDGF-AB, and TGF-β1). (**A**) release per time interval; (**B**) cumulative release up to 14 days. Data from eight participants. *p < 0.05.

The highest amount of VEGF was released by A-PRF-DUO at 1–3 days (2241.4 pg/mb, 95%CI 917.8–3565.0). No statistically significant differences could be observed among any of the membranes (different device/different setting) (*p* > 0.05). For PDGF-AB, the maximum concentration of protein released occurred during the 1–3 day time interval for all membranes. However, no statistically significant difference amongst all membranes was observed (*p* > 0.05). All membranes produced the highest amount of TGF-β1 at the 1–3 day time interval. Statistically significant difference could only be observed between A-PRF-DUO and A-PRF-IL in favour of the latter (*p* < 0.05). Similarly, all membranes produced the highest amount of BMP-1 at a 4 h-1-day time interval. No significant differences could be found between the membranes (*p* > 0.05).

#### Cumulative measurement on release of growth factors (Fig. 1)

The highest amount of VEGF over 14 days was released by L-PRF-DUO (6306.8 pg/mb, 95%CI 1351.3–9075.6). However, no statistically significance difference was found in comparison with other membranes (*p* > 0.05). For PDGF-AB, the highest amount was released by A-PRF-IL (83,692.3 pg/mb, 95%CI 63,976.9–103,407.7), but no statistically significant difference could be found between all membranes (*p* > 0.05). A-PRF + -DUO released the highest amount of TGF-β1 after 14 days (177,974.1 pg/mb, 95%CI 136,761.3–2,191,896.9). This did not reach statistical significance compared with the other membranes (*p* > 0.05). Likewise, A-PRF-IL produced the highest amount of BMP-1 after 14 days (1723.4 pg/mb, 95%CI 731.5–4033.5) but no differences could be observed among any of the membranes (*p* > 0.05).

Women released slightly more growth factors compared with men except for VEGF (L-PRF-IL), PDGF (L-PRF-IL and L-PRF-DUO), and TGF- β1 (L-PRF-IL and A-PRF ^+^-IL), albeit not statistically significant (*p* < 0.05).

#### Cellular counting

**Figure 2:**
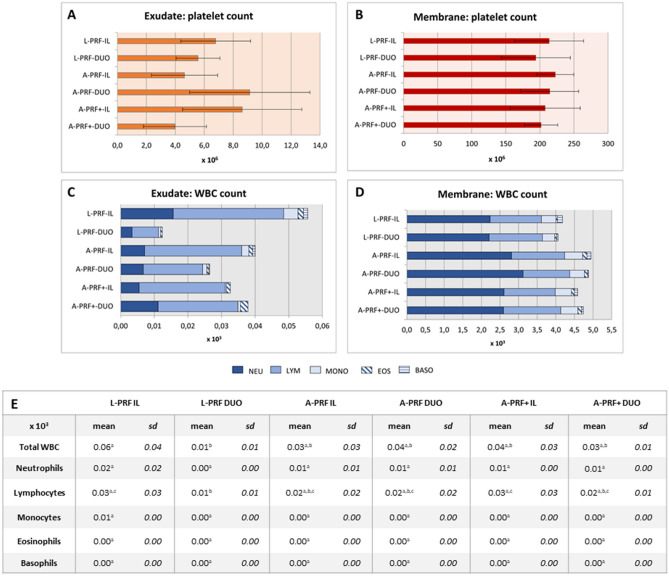
Cellular counting of the exudate and the membranes for each protocol. (**A**) Platelet count for exudate; (**B**) platelet count for membrane; (**C**) white blood cell count for each exudate; (**D**) white blood cell count for each membrane; (**E**) mean and standard deviation (*sd*) of the white blood cells count for the exudate. For each cell type, different letters (a–c) indicate statistical significant difference (*p* > 0.05) between protocols.

All membranes contained more than 60% of leucocytes available in the initial blood sample. No statistically significant differences were observed for any leucocyte cell type amongst protocols (*p* > 0.05). Similarly, all membranes presented more than 80% of platelets, except L-PRF-DUO (74.0%) and A-PRF + -DUO (76.9%). These differences did not reach statistical significance (Fig. 2).

The exudate showed a low cellular content with less than 3% of platelets and 1% of leucocytes for all settings. No statistically significant differences were observed for the platelet count (*p* > 0.05). Statistically significant differences amongst white blood cells are shown in Fig. 2E.

#### Macroscopic analysis (Table 1)

**Table 1:** Clots and membrane dimensions (mean and standard deviation) in all protocols (n = 8).

	Length (cm)		Width (cm)		Weight (g)	
	Mean	*sd*	Mean	*sd*	Mean	*sd*
**Clot**						
L-PRF-IL	3.0	0.3	1.2	0.08	1.6	0.5
L-PRF-DUO	3.3	0.7	1.2	0.1	2.1	0.6
A-PRF-IL	2.9	0.3	1.2	0.1	1.6	0.3
A-PRF-DUO	3.0	0.3	1.1	0.1	2.0	0.3
A-PRF + -IL	2.9	0.3	1.1	0.1	1.5	0.3
A-PRF + -DUO	3.0	0.2	1.1	0.1	1.7	0.2
**Membrane**						
L-PRF-IL	2.9	0.7	1.0	0.1	0.26	0.09
L-PRF-DUO	3.2	0.5	1.0	0.1	0.27	0.08
A-PRF-IL	2.8	0.4	1.0	0.06	0.22	0.08
A-PRF-DUO	3.0	0.4	1.0	0.07	0.27	0.06
A-PRF + -IL	3.0	0.2	0.9	0.1	0.23	0.08
A-PRF + -DUO	2.9	0.5	0.9	0.1	0.22	0.06
**Difference membrane-clot**						
L-PRF-IL	− 0.08	0.6	− 0.2	0.1	− 1.3	0.4
L-PRF-DUO	− 0.09	0.2	− 0.2	0.3	− 1.8	0.6
A-PRF-IL	− 0.1	0.2	− 0.2	0.2	− 1.4	0.2
A-PRF-DUO	− 0.08	0.6	− 0.1	0.6	− 1.7	0.2
A-PRF + -IL	0.06	0.2	− 0.2	0.2	− 1.3	0.2
A-PRF + -DUO	− 0.1	0.3	− 0.2	0.2	− 1.5	0.3

Difference (Δ) between clots and membranes in terms of length, width, and weight. *sd:* standard deviation.

**Figure 3:**
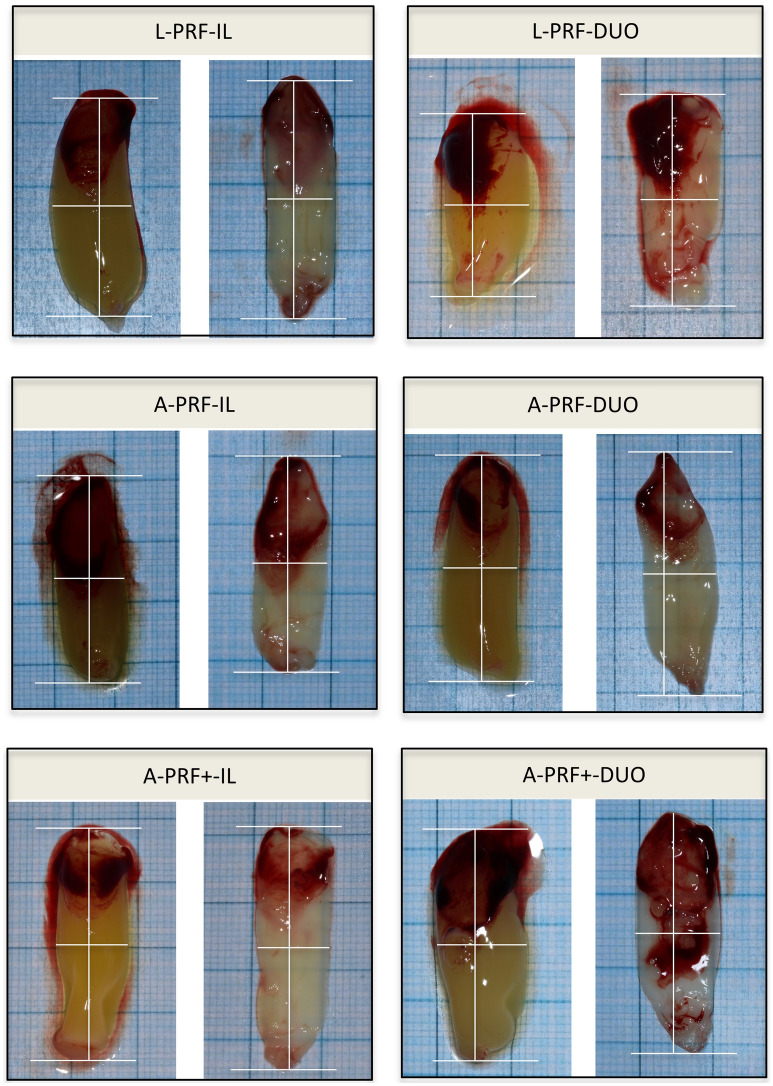
Standardized pictures and measurements of the clots and membranes. The length and the width of each clot and membrane were measured with a horizontal (length) and a vertical (width) line drawn from the middle point of the clot or membrane with an angle of 90°.

In terms of length or width no statistically significant differences could be observed among the different clots. In terms of weight statistically significant differences (1.6 ± 0.4 g vs. 2.1 ± 0.6 g, *p* < 0.05) were only observed between L-PRF-IL clots versus L-PRF-DUO clots. No differences in length, width, or weight were observed in the membranes (*p* > 0.05) (Fig. 3).

After gentle compression of the clot in membranes, a minimal change in length of 0.08 cm, 0.09 cm, 0.10 cm, 0.08 cm, 0.06, and 0.10 cm was measured for L-PRF-IL, L-PRF-DUO, A-PRF-IL, A-PRF-DUO, A-PRF + -IL, and A-PRF + -DUO, respectively. At the same time, a mean loss of 85.5% in weight was recorded (L-PRF-IL: 81.7%, L-PRF-DUO: 87.1%, A-PRF-IL: 84.2%, A-PRF-DUO: 87.2%, A-PRF + -IL: 86.1%, and A-PRF + -DUO: 86.6%).

#### Mechanical testing

**Figure 4:**
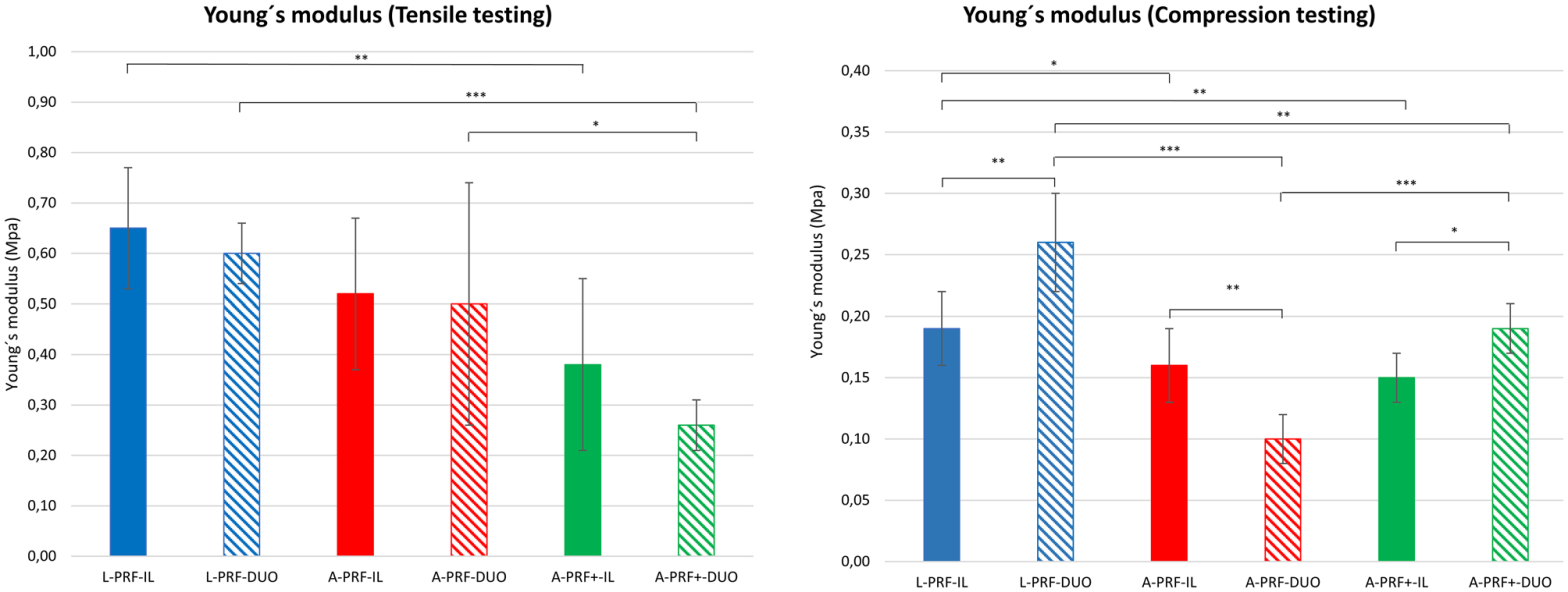
Graphical representation of the compression tests and tensile tests. Data from eight participants. *p < 0.05, **p < 0.01, ***p < 0.001.

Figure 4 shows Young’s modulus obtained for the different membranes (mean and standard deviation).

In the tensile test, statistically significant differences were observed between L-PRF-IL versus A-PRF-IL (*p* < 0.01), L-PRF-DUO versus A-PRF + -DUO (*p* < 0.001), and A-PRF-DUO versus A-PRF + -DUO (*p* < 0.05). No statistically significant difference was found within the same protocol when the g force was adapted in each device, i.e. L-PRF-IL versus L-PRF-DUO.

For the compression test, statistically significant differences were observed between the different centrifugation protocols and within the same protocol using different devices (Fig. 4).

### Influence of time in the preparation

#### Timing: blood draw—centrifugation

**Figure 5:**
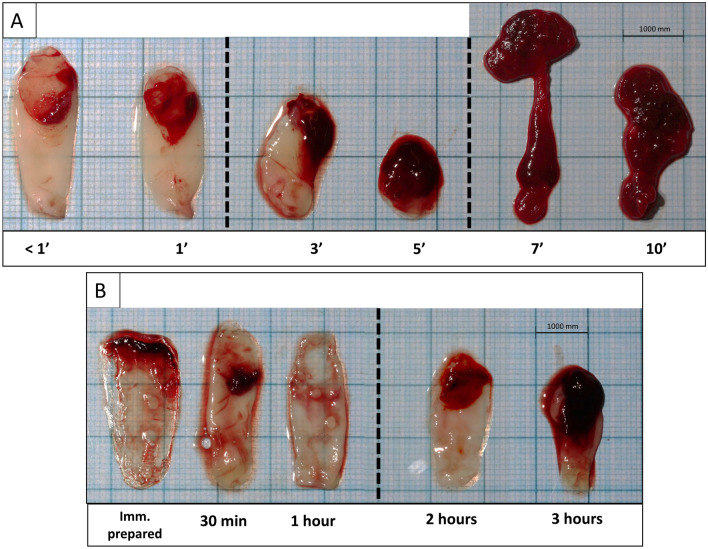
Morphology of the L-PRF membranes depending on the time before and after centrifugation (n = 3). (**A**) Timing between blood collection and centrifugation; (**B**) Timing between centrifugation and L-PRF membrane preparation.

The time interval between blood draw and centrifugation played a significant role. If the delay was > 5 min (in one patient even > 3 min) an amorphous blood clot was obtained and compression into a membrane became impossible (Fig. 5). Even shorter delays had an impact. For example, delays caused a reduction in membrane length: for time intervals of < 1, 1, 3, and 5 min lengths were 3.0 ± 0.2 cm, 2.4 ± 0.5 cm, 1.7 ± 0.1 cm, and 0.9 ± 0.0 cm, respectively). The difference between < 1 or 1 min on the one hand and 3 min or 5 min on the other hand reached statistical significance (*p* < 0.05). No statistical significant difference could be observed for width measurements (*p* > 0.05).

**Figure 6:**
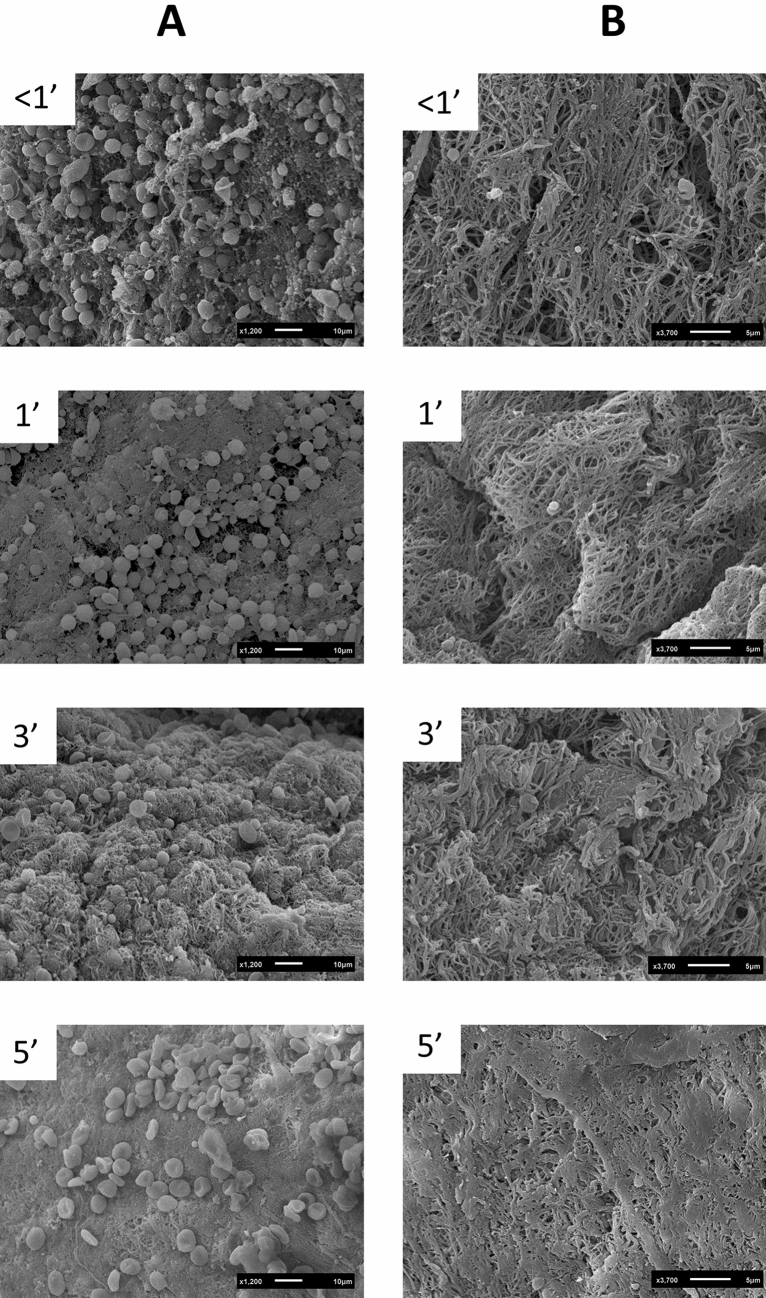
Scanning electron microscopy images of the L-PRF membranes with different time points (< 1 min, 1 min, 3 min, and 5 min) before centrifugation. (**A**) Represents the red part (face) of the membrane; (**B**) represents the yellow part (tail).

SEM images from the above-mentioned membranes are shown in Fig. 6. Membranes prepared within 1 min after blood draw showed clusters of platelets, leucocytes and red blood cells embedded in a well-organized fibrin matrix. As the time interval increased, looser cells and a denser and disorganized matrix were observed.

#### Timing: centrifugation—membrane preparation (Fig. 5)

The time interval between the end of centrifugation and the compression of the clot into a membrane also had an impact. The longer this time interval, the smaller the membranes. Both in length and width, statistically significant differences were found between membranes prepared immediately after centrifugation and those prepared after 2 or 3 h (*p* < 0.05).

## Discussion

The present study showed both the importance of adapting RCF of the centrifuge to obtain similar PRF matrices and the differences among different PRF modifications. No statistically significant differences could be observed among all six protocols in terms of growth factors release, cellular content, and dimensions. The release of growth factors by L-PRF, A-PRF and A-PRF ^+^ had already been reported in literature, but to date PRF clots had been examined and not the membranes^13,16^. Ours is the first study to test PRF membranes. Choosing membranes rather than clots is important because membranes are most frequently used in oral surgery and treatment of chronic wounds^17–19^.

The role of growth factors in bone formation is widely recognised, particularly for bone morphogenetic proteins (BMPs), PDGF, TGF-β and VEGF^20,21^. BMPs and PDGF induce migration and proliferation of osteoprogenitor cells, whereas TGF-β stimulates cell growth and the synthesis of extracellular matrix^22^. VEGF is known as a potent inductor of angiogenesis and osteoblast proliferation^23^. Recently, Ratajczak and co-workers^24^ described the angiogenic potential of L-PRF in an *in-vitro* study. They concluded that L-PRF induced key steps of the angiogenic process such as endothelial proliferation, migration, and tube formation. They also demonstrated that L-PRF was able to induce blood vessel formation in vivo with a chorioallantoic membrane assay. Platelet concentrates may release other bioactive factors that also play a role in the regeneration process. Some studies have revealed that PRF constructs produced key immune cytokines, such as interleukin (IL) 1β, IL-6, IL-4, and tumor necrosis factor^25,26^.

Several papers^13,15,27^ reported contradictory data on the impact of *g*-force on the above-mentioned release in growth factors, but unfortunately with some methodological shortcomings^7,28^. It is indeed important to use the same RCF of the centrifuge when comparing different protocols. When adapting RCF for different centrifuges, the previously reported differences between different protocols were no longer observed. Indeed, in the current study, no statistically significant differences could be found among all membranes, prepared with different protocols, in terms of growth factors release, cellular content, and dimensions.

In literature, the common way to express rotor speed is in terms of revolutions per minute (rpm)^29,30^. However, rpm does not take into account the radius of the centrifuge. Since the radius is not standardized for all centrifuges, RCF should be used because it also considers the distance of the tubes to the axis of rotation^31^. Therefore, the rpm of two centrifuges can be the same, but the forces applied to the particles in the tubes will differ significantly. Both the Intra-Spin and the PRF DUO centrifuge have a fixed-angle rotor but they present a different rotation angle and radius (Table 2). Consequently, RCF should be adjusted in both centrifuges if one would like to apply similar forces on the blood cells. If not, PRF constructs will be completely different. One limitation of this study is that the angulation of the tubes inside the centrifuge is a variable that cannot be adapted. Thus, we assumed that the angulation would have some effect on the distribution of the cells in the clot/membrane. However, even without adapting this variable, no differences could be observed among all membranes.

**Table 2:** Platelet rich fibrin matrices (L-PRF, A-PRF, and A-PRF +) prepared with the centrifuge process for PRF, Nice, France, (DUO)] and the Intra-Spin centrifuge [Intra-Lock, Biohorizons, Birmingham, Alabama, USA, (IL)]^7^.

	Device	Tube	Setting				
			RCF_clot_ (g)	RCF_max_ (g)	Rotor angulation (°)	rpm	Time (min)
L-PRF-IL	Intra-Lock, USA	Plastic-coated	408	653	33	27,000	12
L-PRF-DUO	Process for PRF, France	Glass	408	653	40	1825	12
A-PRF-IL	Intra-Lock, USA	Plastic-coated	193	276	33	2200	14
A-PRF-DUO	Process for PRF, France	Glass	193	276	40	1500	14
A-PRF + -IL	Intra-Lock, , USA	Plastic-coated	145	208	33	1900	8
A-PRF + -DUO	Process for PRF, France	Glass	145	208	40	1300	8

The L-PRF clot lost 85% of its weight when compressed into a membrane. However, the clots were compressed using a box where the force applied could not be registered, which may be a limitation of this study. Nevertheless, this finding needs to be taken into consideration when using L-PRF clots in a clinical application, for instance, for alveolar ridge preservation. If clots are used, it will be more difficult to place 4–6 clots in one socket since their volume is larger than that of the membranes^32,33^. After some time, the clots will lose the exudate, leaving an almost empty socket. The latter might explain the unfavourable clinical results when clots were used for ridge preservation instead of membranes^34^.

Although there was no statistically significant difference, PRF clots produced with glass tubes showed higher weight (mean 1.9 ± 0.4 g) compared with those from silica-coated plastic tubes (mean 1.6 ± 0.3 g). Recently, the importance of centrifugation tubes on the final production of PRF matrices has been highlighted^35^. Bonazza and co-workers^36^ already showed the influence of the material and shape of the blood collection tube on the platelet concentrate, with differences in morphology, fibrin network architecture, and cell distribution. They reported a higher weight of Concentrated Growth Factors (CFG), a PRF-like product, obtained with a glass round-bottom tube. In glass tubes, blood begins to coagulate immediately after blood collection, with larger fibrin recruitment. The use of these tubes allowed to obtain CFGs that were larger, thicker and weighed more compared with those obtained with plastic silica-coated tubes^36,37^. However, their weight after compression, in membrane form, did not differ between protocols in our study. Yamaguchi and co-workers^38^ reported different platelet distribution in the concentrated growth factors matrix when prepared with silica-coated plastic tubes or glass tubes. Platelets were distributed mainly in the distal side of the glass-prepared CGF matrix, but homogeneously in the plastic-prepared CGF matrix.

In this present study, statistically significant differences were found amongst different protocols for both mechanical tests. Moreover, the results for the tensile testing were similar and showed no statistically significant differences for the same protocol when the *g*-force was adapted in both centrifuges, suggesting that the adaptation of the *g*-force may result in similar PRF matrices independently of the device used. The results obtained from the mechanical testing for the six protocols ranged between 0.3–0.6 MPa and between 0.1–0.3 MPa for the tensile strength and compression strength, respectively. These data are similar to the results found in literature^39,40^. For instance, when compared with the elastic modulus in tension of a porcine dermal collagen membrane (0.3 ± 0.1 MPa)^41^, all PRF constructs showed higher values except A-PRF + -IL and A-PRF + -DUO.

In this study, and for the first time, the relevance of timing before and after centrifugation in obtaining an optimal PRF construct is highlighted. The blood coagulation cascade has been studied intensively^42–44^. Butenas and co-workers^45^ reported that this first stage of the coagulation finished after 2 min. The *in-vivo* clot formation has also been studied with real-time confocal microscopy^46^. After 20 s, fibrin appeared on the upstream edge of the thrombus. Between 34 and 60 s, fibrin extended throughout the platelet thrombus. Accordingly, our findings showed the importance of centrifuging blood within the first 60 s to avoid early formation (before centrifugation and separation of the cells) of a coagulum inside the tube. These findings are in accordance with those observed by Miron and co-workers^47^. They also reported a 60- to 90 s interval between blood draw and the start of centrifugation.

Within the limitations of this study, one can conclude that the adaptation of RCF for each centrifuge did not result in differences in terms of release of growth factors, cellular content, dimensions, and mechanical properties. However, the time between blood collection and centrifugation strongly influenced the dimension and structure of the L-PRF membranes obtained.

## Methods

Eight healthy volunteers were included in this study. The exclusion criteria comprised the following conditions: anticoagulant medication 3 months before the study, pregnancy or lactation, history of periodontal disease or any active systemic infection. A total of six 9-ml silica-coated plastic tubes (BVBCTP-2, Intra-Spin, Intra-Lock, Florida, USA), and six 10-ml glass tubes (A-PRF tubes, Process for PRF, Nice, France) were collected per participant. Three out of eight volunteers donated an extra 12 tubes of blood for the timing experiments: six for the blood draw—centrifugation time (time before centrifugation), and six for the centrifugation—membrane preparation time (time after centrifugation). The tube distribution for each experiment is shown in Fig. S1.

The use of human blood was approved by the KU Leuven ethical committee and registered with identifier B322201628215. The procedures were executed according to the Helsinki Declaration and the regulations of the University Hospital, which are approved by the ethical committee. An informed consent was obtained from all subjects.

### Comparison of PRF modifications

#### Preparation of the PRF clots/membranes

Three types of platelet concentrates were prepared with two different centrifuges and the *g-*force was adapted for each protocol: leucocyte- and platelet-rich fibrin (L-PRF) (RCF_clot_ 408 g, RCF_max_: 653 g, for 12 min)^33^; advanced platelet-rich fibrin (A-PRF) (RCF_clot_ 193 g, RCF_max_: 276 g for 14 min)^11^; and advanced platelet-rich fibrin + (A-PRF +) (RCF_clot_ 145 g, RCF_max_: 208 g for 8 min)^12^.

These clots were gently compressed into membranes using the specific design box for each protocol (L-PRF: Xpression kit, Biohorizons, Birmingham, Alabama, USA ; A-PRF and A-PRF + : PRF Box, Process for PRF, Nice, France). Two centrifuges were used, in which the *g*-force could be adapted: the DUO centrifuge [Process for PRF, Nice, France, (DUO)] and the Intra-Spin centrifuge [Biohorizons, Birmingham, Alabama, USA, (IL)].

Six different membranes were prepared: L-PRF-DUO, A-PRF-DUO, A-PRF + -DUO; and L-PRF-IL, A-PRF-IL, A-PRF + -IL (Table 2). Following the manufacturer’s instructions, 10-ml glass tubes were used for all the preparations in the DUO centrifuge, and 9-ml silica-coated plastic tubes for the Intra-Spin centrifuge.

#### Release of growth factors

Each membrane (L-PRF-DUO, L-PRF-IL, A-PRF-DUO, A-PRF-IL, A-PRF + -DUO, A-PRF + -IL) was placed in a 15 ml-tube with 5 ml of Dulbecco’s Modified Eagle medium (Sigma-Aldrich BVBA, Overijse, Belgium) without antibiotics and changed to a new tube with sterile tweezers after 4 h and subsequently on day 1, 3, 7, and 14. After membrane collection at each time interval, the remaining medium was centrifuged at 1000 rpm for 10 min (VWR Mega Star 6000R, VWR International BVBA, Haasrode, Belgium) to remove any residue. Next, the medium was frozen at -80° Celsius.

Following the protocol described by Castro and co-workers^48^, the concentrations of platelet-derived growth factor-AB (PDGF-AB), transforming growth factor beta-1 (TGF-β1), and vascular endothelial growth factor (VEGF) were calculated in duplicate with commercially available enzyme-linked immunosorbent assay kits (ELISA, R&D Systems Europe, Abingdon, UK) following the manufacturer’s instructions. The levels of bone morphogenetic protein-1 (BMP-1) were also recorded by another ELISA test (Abbexa, Cambridge Science Park, UK). Measurements were conducted with a microplate reader (Multiskan Ascent, Rev 1.2, Thermo Electron Corporation, Vantaa, Finland) set to 450 nm and using 550 nm as a background reference. The exudate released during compression of the clot into a membrane was kept to analyse the cellular content.

#### Cellular counting

**Figure 7:**
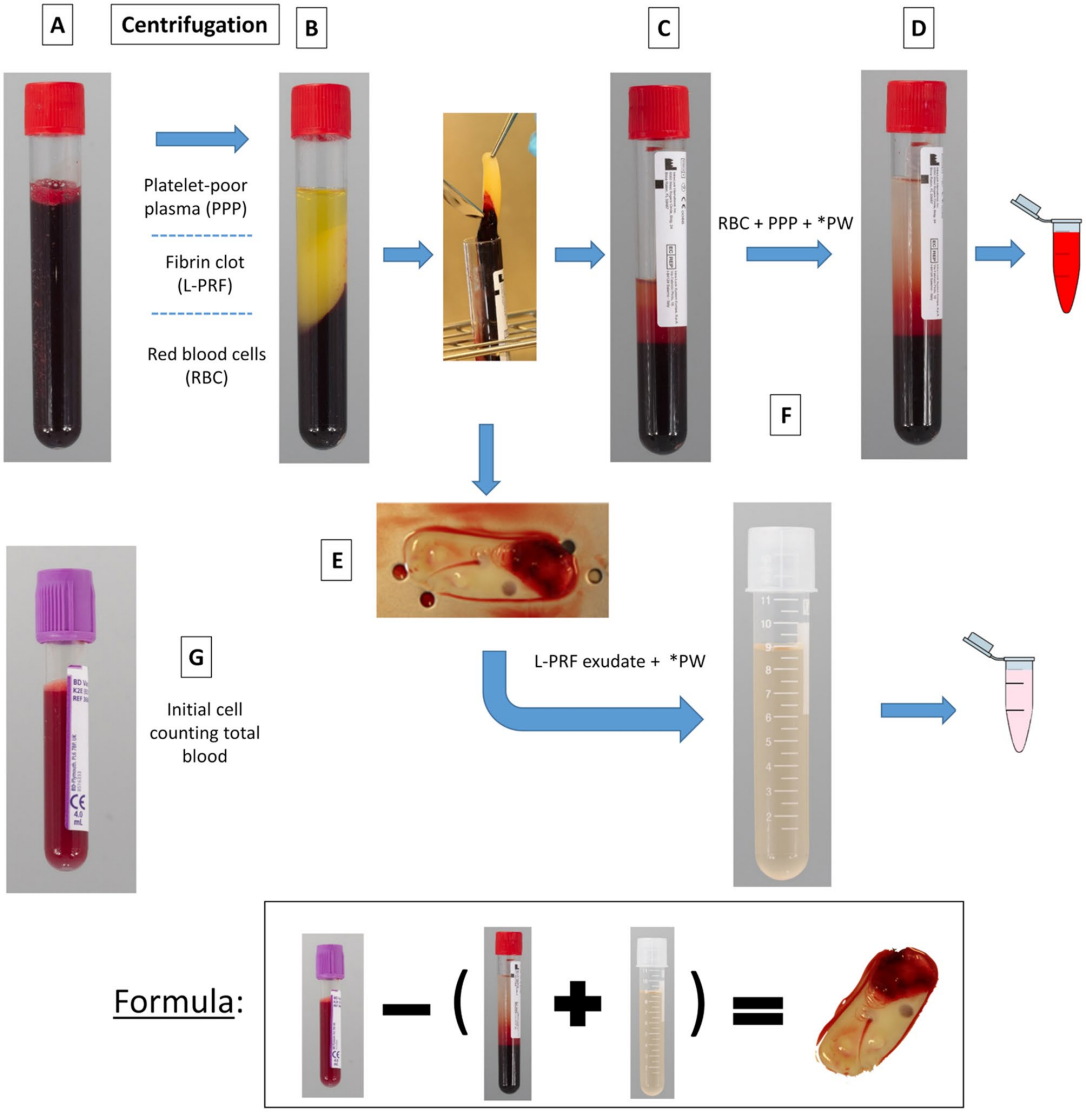
Representation of steps for cellular counting. (**A**) initial blood; (**B**) tube after centrifugation with three layers: platelet-poor plasma (PPP), fibrin clot (L-PRF clot), and red blood cells (RBC); (**C**) tube after removal of the fibrin clot (PPP + RBC). (**D**) same tube of step C with physiological water (PW) until a volume of 9 ml (initial volume); (**E**) L-PRF membrane prepared after compression of the clot; (**F**) tube with L-PRF exudate release during the compression of the L-PRF clot + physiological water (PW) until a volume of 9 ml; (G) tube with initial blood composition.

Cellular counting was performed for all the membranes with a haematology analyser (CELL-DYN 3700, Abbott GmbH & Co, Wiesbaden, Germany). Given the difficulty to dissolve the membranes without damaging the cells, cellular counting was carried out indirectly following the protocol described by Castro et al.^48^ (Fig. 7). All samples were frozen at -80° Celsius after addition of 10% dimethylsulfoxide (DMSO) to avoid the formation of crystals inside the cells.

#### Macroscopic analysis

After centrifugation, the clots were removed from the tubes and weighed immediately. Standardized pictures were taken of all the clots on a graph paper. The clots were then transformed into a membrane by gentle compression. Membranes were weighed and standardized pictures taken. The length and the width of each clot and membrane were measured with software ImageJ (Image Processing and Analysis in Java, 1.8.0_77). A horizontal (length) and a vertical (width) line were drawn from the middle point of the clot or membrane with an angle of 90°.

#### Physical characteristics

Tensile tests were carried out on a TA.XT plus Texture Analyser (Stable Microsystems, Surrey, UK). The membranes had previously been cut with a specially designed metal mould with a “dog-bone” shape. The dimensions were 5 mm width in the narrowest middle part, 10 mm width at both ends, and 1 mm in thickness. The shaped membranes were held with the tensile grips of the Texture Analyser (A/MTG Mini), leaving the specimen free of tension. Next, the test was programmed on Exponent software (Stable Microsystems, Surrey, UK) applying the tensile load at a constant speed of 0.5 mm/s. Stress–strain curve data were recorded and the elastic modulus was calculated by using the slope of the stress/strain curve.

For the compression test, the same equipment was used but the membranes were cut with a 10-mm diameter metal punch, and the tensile grips were changed by a cylinder probe P/0.5 (Stable Microsystems, UK). The specimens were compressed to about 50% (50% deformation) at a constant speed of 0.5 mm/s. Stress–strain curve data were recorded and the elastic modulus was calculated by using the slope of the stress/strain curve.

### Influence of time in the preparation of platelet concentrates

#### Timing: blood draw—centrifugation

Six extra 9-ml plastic silica-coated tubes were collected from three participants. One tube was centrifuged immediately (< 1 min) at 408 g for 12 min, whereas the remaining five were gently shaken for 1, 3, 5, 7, and 10 min before centrifugation. Standardized pictures were taken of the corresponding membranes. Morphology (length and width) was measured with ImageJ as described for the macroscropic analysis.

One L-PRF membrane from each time point (if a membrane could be obtained) was processed for SEM analysis following the protocol described by Castro and co-workers^48^ (2019). Briefly, each membrane was fixed immediately after preparation in 2.5% glutaraldehyde in 0.1 M sodium cacodylate buffer for 24 h, rinsed with 0.2 M sodium cacodylate buffer and distilled water, and dehydrated in ascending dilutions of ethanol (25, 50, 75, 95, and 100%). After dehydration, each sample was immersed in hexamethyldisilazane 98% (Acros Organics, Geel, Belgium) for 10 min and air-dried at room temperature. The specimens were coated with gold by an auto fine coater (JFC-1300, JEOL, Tokyo, Japan). The images from the red part (face, the area previously in contact with the red blood cells) and the middle part (tail) were taken using SEM (JSM-6610LV, JEOL).

#### Timing: centrifugation—membrane preparation

Another six 9-ml plastic silica-coated tubes from 3 patients were centrifuged immediately at 408 g for 12 min. L-PRF membrane were prepared immediately after centrifugation and after 30 min, 1 h, 2 h, and 3 h, respectively.

### Data analysis

In order to analyse growth factor release, the fixed effects coefficients and their variance–covariance matrix were subjected to a multiple comparisons procedure with the best^49^. The concentrations of growth factors for each membrane were calculated, per time interval and cumulative concentration.

A linear mixed model was set up for each haematological cell type separately, with each protocol as fixed factor and the subject as random factor. Normality of the residuals was assessed by means of a normal quantile plot and data were log-transformed if the normal quantile plot indicated a distribution that approached a log-normal distribution.

The data for the mechanical testing were analysed using descriptive statistics by reporting the mean and standard deviation. An unpaired student t-test was used to compare all groups.

For the data of the time before/after centrifugation, a linear mixed model was applied with donor as random factor. A normal quantile plot was used to assess the normality of the residuals. Comparisons between timings were corrected for simultaneous hypothesis testing according to Tukey.

## Supplementary Information

Supplementary Information.
