# Supplementary figures and images for: Impact of g force and timing on the characteristics of platelet-rich fibrin matrices
Source: Sci Rep. 2021 Mar 16;11:6038. doi: 10.1038/s41598-021-85736-y (PMC7971031; doi:10.1038/s41598-021-85736-y)

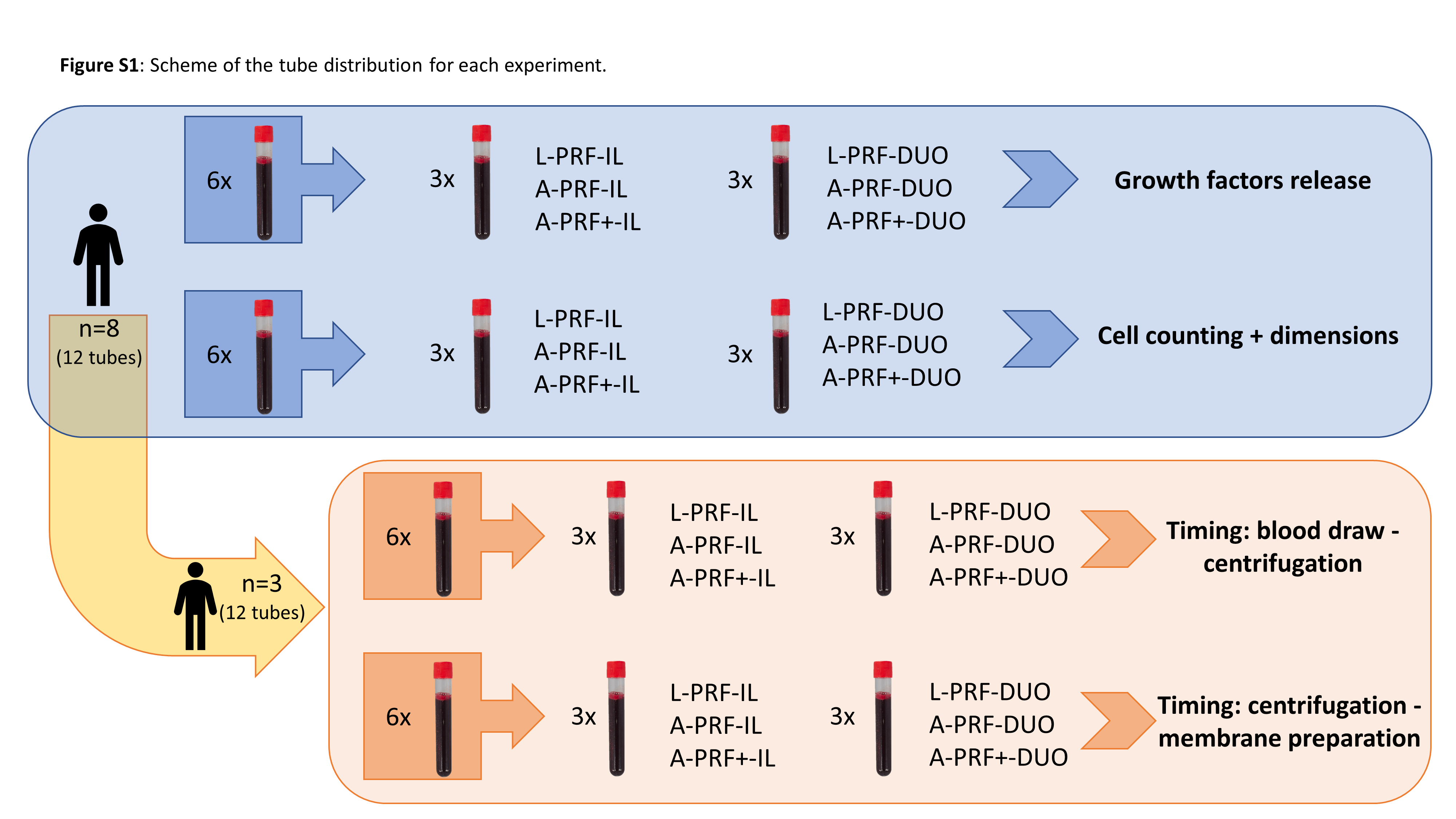

Supplement: Supplementary file 1 — Supplementary Information. [file 41598_2021_85736_MOESM1_ESM.tif]
